# Hepatic Epithelioid Angiomyolipoma Treated with Laparoscopic Resection: Case Report and Review of the Literature

**DOI:** 10.1155/2019/2362618

**Published:** 2019-07-01

**Authors:** Chloe H. Williams, Kelli Hickle, Katherine Bakke, Sarah Jamshed, Adel Bozorgzadeh

**Affiliations:** ^1^UMass Memorial Medical Center, Department of Surgery, Worcester, MA 01655, USA; ^2^UMass Memorial Medical Center, Department of Pathology, Worcester, MA 01655, USA

## Abstract

Hepatic angiomyolipoma is a rare primary liver tumor, with a radiographic appearance very similar to hepatocellular carcinoma. We present the case of a noncirrhotic patient with a liver tumor suspicious for HCC by imaging features. Liver biopsy demonstrated angiomyolipoma, and the patient successfully underwent a laparoscopic liver resection.

## 1. Introduction

Hepatic angiomyolipoma (AML) is a rare primary liver tumor, described only by small case series. Most cases occur in middle-aged women, who either are asymptomatic or describe vague abdominal pain. AMLs are tumors of mixed mesenchymal origin and predominantly occur in the kidney. Radiographically, hepatic AML can be difficult to differentiate from other liver tumors, particularly hepatocellular carcinoma (HCC), as they commonly appear as well-defined lesions with CT arterial phase enhancement and “washout” in the portal and delayed phases. Imaging features are not specific and can lead to a misdiagnosis of HCC if pathologic confirmation is not obtained [1]. The gold standard for the diagnosis of hepatic AML is histologic examination and immunohistochemical staining from biopsy or surgical specimen.

We present a case of epithelioid hepatic AML diagnosed preoperatively by biopsy and successfully laparoscopically resected.

## 2. Case Report

A 54-year-old Caucasian female with a history of cholecystectomy, obesity, and hypertension presented to the Emergency Department with acute onset right upper abdominal pain. Basic laboratories including liver function tests, complete blood count, and basic metabolic panel were unrevealing.

**Figure 1:**
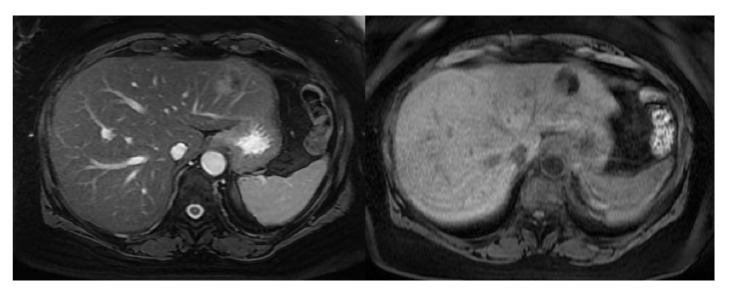
*MRI*. FIESTA (right) and T1-weighted images (left) revealed a 3.1 x 3.4 x 2 cm mass in the left lateral lobe of the liver. The lesion is hypervascular with washout of contrast on delayed post-contrast enhanced sequences.

Outpatient workup revealed normal AFP, CA19-9, and CEA levels and negative hepatitis serologies. Ultrasound demonstrated a 2.3 cm, low-density mass in the left liver lobe. MRI showed a 3.4 cm mass with arterial phase enhancement and contrast washout in delayed sequences (Figure 1). By imaging, a diagnosis of HCC was suspected. However, as she did not have cirrhosis or other risk factors for HCC, she underwent percutaneous liver biopsy and was diagnosed with epithelioid AML.

The patient underwent laparoscopic hand-assisted left lateral hepatic segmentectomy. Our surgical approach included three trocar sites and a small midline hand port. Resection was completed using a combination of Ligasure and Cavitron Ultrasonic Surgical Aspirator (CUSA). One Jackson-Pratt drain was left at the cut edge of the liver.

**Figure 2:**
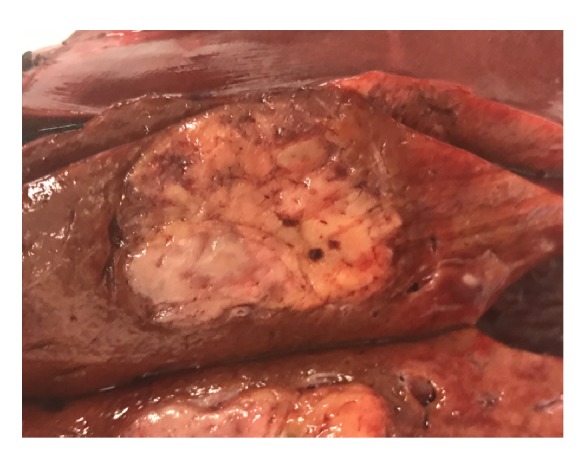
*Gross specimen*. On gross examination of the liver specimen, the cut surface of liver showed a well-demarcated, glistening, tan-yellow, lobular mass, measuring 3.0 x 2.5 x 2.0 cm. Final surgical pathology confirmed the diagnosis of epithelioid angiomyolipoma with no evidence of malignancy and 2.2 cm surgical margins.

**Figure 3:**
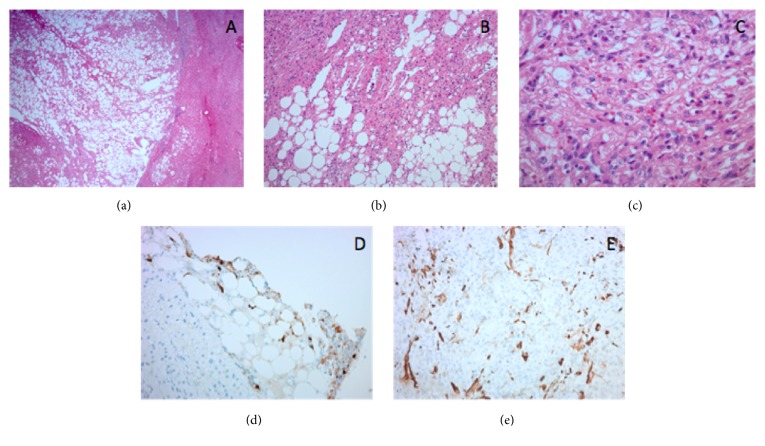
*Histopathologic features of epithelioid angiomyolipoma*. (a) Well-circumscribed mass with adjacent, mildly steatotic liver parenchyma (H&E; 20x). (b) The tumor is composed of eosinophilic cells, mature adipocytes, and thick walled vessels (center) (H&E; 100x). (c) The smooth muscle component includes a mixture of large epithelioid cells with abundant eosinophilic cytoplasm and prominent nucleoli, with interspersed smaller epithelioid cells and spindle cells (H&E; 400x). Immunohistochemistry performed on the biopsy of this lesion for HMB45 (200x) (d) and Smooth Muscle Actin (200x) (e) showed patchy, strong positivity in the lesional cells.

Pathologic gross examination revealed a 3.0 x 2.5 x 2.0 cm mass, well-demarcated from adjacent hepatic parenchyma (Figure 2). On microscopy, an admixture of haphazardly arranged mature adipocytes, smooth muscle fascicles, and thick-walled vessels was seen, which was positive for HMB45, MelanA, MITF, and smooth muscle actin and negative for pan-cytokeratin, HepPar1, and S100, confirming the diagnosis (Figure 3). She was discharged after an uneventful hospital course.

The patient was seen in follow-up one year after her tumor resection. She was doing well and had fully returned to her presurgical level of function. 4-phase liver CT done at that time demonstrated no tumor recurrence or evidence of abdominal metastasis. LFTs were all within normal range.

## 3. Discussion

Hepatic AML typically presents as an incidentally found liver mass or in patients with vague abdominal discomfort, as in our case. Rarely are these tumors large enough to lead to presentation from mass effect, palpation, or rupture, though this has all been reported [2]. There is a known link associated with tuberous sclerosis of 5-15%, but this link is stronger in renal AML [3]. Hepatic epithelioid AML is rare and routinely misdiagnosed. One report identified 292 patients with hepatic AML, only 12 of which were epithelioid subtype [4].

On contrast-enhanced imaging, both hepatic AML and HCC appear hyperenhanced in the arterial phase with early washout times [5]. Several reports reveal rates of misdiagnosis of hepatic AML as HCC ranging from 25 to 80% [1, 5–7]. Because radiologic features make hepatic AML difficult to distinguish from HCC, the diagnosis of hepatic AML relies on immunohistochemical staining; HMB45 and SMA are positive, and Hep Par1 and S100 are negative [8]. On histology, smooth muscle presence with variable distribution of epithelial cells, adipose cells, and blood vessels is seen.

Misdiagnosis as HCC has serious clinical and psychological implications for patients. Biopsies are not routinely performed once a radiographic diagnosis of HCC is made. Traditional treatment for HCC in a noncirrhotic patient is liver resection. However, liver transplantation is evolving as the standard of care for small HCCs due to simultaneous survival benefit and reduction in disease recurrence [9]. The diagnosis of HCC in our otherwise healthy patient was suspicious in the absence of other risk factors; therefore image-guided biopsy was performed to confirm her diagnosis. Preoperative biopsy providing accurate diagnosis in this case was useful as it allowed for comprehensive patient education and surgical planning. With liver transplant becoming an option for HCC management in many patients, misdiagnosis by imaging alone could have led to unnecessary and invasive procedures. Due to the risk of malignant transformation or rupture of hepatic AML [10], resection is typically recommended. In select cases, a nonoperative approach with close surveillance is appropriate as mortality remains low at 0.8% [4].

Accurate preoperative diagnosis as AML allowed the patient and surgeon to come to an informed decision and to undergo the appropriate treatment of minimally invasive resection. Damaskos et al. reported the first laparoscopic resection of hepatic AML in 2017, and our case further demonstrates the safety and utility of this approach [11]. In the absence of metastatic lesions, most reports support good surgical outcomes and long-term survival for hepatic AML patients [2, 3].

We encourage clinicians to have a high index of suspicion of misdiagnosis of HCC in noncirrhotic patients without other HCC risk factors and to employ preoperative biopsy to confirm diagnosis in these cases. A correct diagnosis is crucial to tailor appropriate tumor management. HCC warrants excision in noncirrhotic patients and, increasingly, referral for transplant evaluation; hepatic AML can be treated with a laparoscopic resection as a safe, novel alternative to open surgery or clinical observation.
